# Interleukin-25 enhances humoral immune responses caused by the rabies virus

**DOI:** 10.1080/21505594.2022.2116146

**Published:** 2022-08-26

**Authors:** Yue Zhang, Mengwei Zhang, Xilan Liao, Yunsong Yu, Qing Liu, Yongwen Luo, Jun Luo, Xiaofeng Guo

**Affiliations:** College of Veterinary Medicine, South China Agricultural University, Guangzhou, China

**Keywords:** Rabies virus, interleukin-25, vaccine, adjuvant, virus neutralizing antibodies

## Abstract

Rabies is an important zoonotic disease caused by the rabies virus (RABV). Currently, no effective treatment is available for this condition. The prevention and control of rabies mainly depend on effective vaccination. Therefore, it is crucial to enhance the immune responses induced by the rabies vaccine. Virus neutralizing antibodies (VNA) induced by rabies vaccines are important for the clearance of RABV. Interleukin-25 (IL-25) has been demonstrated to activate T helper type 2 cells that contribute to humoral immune responses. The IL-25 gene was inserted into the genome of RABV, and the immunogenicity of recombinant RABV with IL-25 gene was investigated to develop more efficient rabies vaccines. Here, we found that the expression of IL-25 did not affect RABV production *in vitro* and pathogenicity *in vivo*. However, recombinant RABV expression of IL-25 induced a better VNA level than the parental virus in mice. In addition, expression of IL-25 enhanced the IgG1 level induced by RABV. Furthermore, mice immunized with recombinant RABV showed a higher survival rate and milder clinical signs than those immunized with the parent strain after challenge with CVS-11. Thus, these results showed that IL-25 could enhance the humoral immune responses induced by RABV, suggesting that IL-25 can be used as a viral vaccine adjuvant.

## Introduction

Rabies is a zoonosis with mainly damages in the central nervous system (CNS) and still an important public health problem worldwide. At least 55,000 humans died after infection by rabies virus (RABV) each year and most cases occurred in the developing countries, especially in Asia and Africa. Dogs with rabies virus are the main infection source [1]. Rabies has a high mortality rate in both humans and dogs without effective treatment. However, rabies vaccines can effectively prevent this medical condition. Therefore, it is crucial to improve the immune responses induced by the vaccine.

Rabies is caused by the RABV. RABV is a nonsegmented, single-stranded, negative-sense RNA virus, belongs to the genus *Lyssavirus* of the family *Rhabdoviridae*. The RABV genome is approximately 12 kb in size. From 3’ to 5,’ the genome of RABV encodes nucleoprotein (N), phosphoprotein (P), matrix protein (M), glycoprotein (G), and an RNA-dependent RNA polymerase (L). RABV has been applied as an excellent vector to express exogenous proteins since the first recombinant RABV was produced [2]. Previous studies have reported that many foreign proteins are expressed between N and P, P and M, and G and L [3–6]. Furthermore, researches have indicated that immunomodulatory molecules expressed between G and L genes significantly enhance the immunogenicity of RABV [3,7]. Therefore, the expression of immune-enhancing molecules in RABV is a good strategy to develop more efficient rabies vaccines.

Interleukin-25 (also known as IL-17E) is a member of the IL-17 family of cytokines based on amino acid sequence homology [8]. IL-25 is secreted by various cells, including CD4+ T cells, CD8+ T cells, macrophages, dendritic cells, mast cells, eosinophils, and epithelial cells [9]. Overexpression of IL-25 increases IL-4 and IL-5 cytokines production, activating Th2 immune responses [10]. IL-25-deficient mice infected with pathogenic microorganisms cannot to eradicate the infection, which coincides with a failure in Th2-type immunity [11,12]. Therefore, IL-25 plays an essential role in infectious diseases.

This study investigated whether IL-25 could increase the immunogenicity of RABV. IL-25 was expressed between G and L genes in RABV, and its immunogenicity was determined in mice. The results indicated that IL-25 expressed in RABV did not inhibit viral replication *in vitro* and enhanced viral immunogenicity *in vivo*.

## Materials and methods

### Cells, viruses, antibodies, and animals

Baby hamster kidney (BHK-21) cells and BSR cells (a clone of baby hamster kidney-21 cells) (Wuhan Institute of Biological Products, Wuhan, China) were maintained in Dulbecco’s Modified Eagle’s Medium (DMEM) (Gibco, Suzhou, China). In addition, mouse neuroblastoma (NA) cells (Wuhan Institute of Biological Products, Wuhan, China) were cultured in Roswell Park Memorial Institute (RPMI) 1640 medium (Gibco, Suzhou, China). Both DMEM and RPMI were supplemented with 10% foetal bovine serum (FBS) (Gibco, Australia). HEP-Flury was rescued from the plasmid pHEP-3.0 (provided by Dr. Kinjiro Morimoto), which contains the full-length cDNA of HEP-Flury in BHK-21 cells, and propagated in NA cells. In addition, challenge virus standard 11 (CVS-11) (a gift from Dr. Xianzhu Xia, Academy of Military Medical Sciences, Beijing, China) was propagated in BHK-21 cells. Fluorescein isothiocyanate (FITC)-conjugated antibodies against RABV N were purchased from Fujirabio Inc. (Malvern, PA, USA). Anti-IL-25 antibody was purchased from Signalway Antibody LLC (Maryland, USA). HRP-conjugated antibodies against IgG1 and HRP-conjugated antibodies against IgG2a were purchased from ABclonal Biotechnology Co., Ltd. (Wuhan, China). Anti-RABV M antibody was produced in our laboratory (unpublished data). Female Kunming (KM) mice were purchased from Zhuhai BesTest Bio-Tech Co., Ltd. (Zhuhai, China). The mice were housed in the Laboratory Animal Center of the South China Agricultural University. The animal experiments were allowed by the Ethics Committee for Animal Experiments of the South China Agricultural University (under the code 2019B190).

## Construction of recombinant full-length cDNA clones and rescue of the viruses

The *mus musculus* IL-25 gene was synthesized (Sangon Biotech Co., Ltd., Shanghai) based on the published sequence on NCBI (NM_080729.3). Recombinant RABV infectious clones were developed by inserting IL-25 gene between G and L genes using restriction sites *Bsi*w I and *Nhe* I with the primers IL-25-forward (5’-AAGCGTACGATGTACCAGGCTGTTGC-3;’ the underlined segment indicates *Bsi*w I restriction site) and IL-25-reverse (5’-AAAGCTAGCCTAAGCCATGACCCG-3;’ the underlined segment indicates *Nhe* I restriction site). Sequences corresponding to IL-25 were inserted into the vector pHEP-3.0, which carried the full-length cDNA of HEP-Flury. The recombinant plasmid was named pHEP-IL-25. Insertion of IL-25 was confirmed by restriction analysis and DNA sequencing. The recombinant RABV expressing IL-25 was rescued from BHK-21 cells [13,14]. The rescued recombinant RABV expressing IL-25 was named rHEP-IL25. The rescued virus was confirmed in NA cells by a direct fluorescent antibody assay (dFA) with FITC-labelled anti-RABV N antibodies, as described previously [15]. The IL-25 and RABV M expression was confirmed by western blotting as described previously [16] using mouse anti-IL-25 and anti-RABV M antibodies. Protein fingerprints were shown using Fine-do 96 (Tanon, China).

## Detection of the recombinant virus with fluorescent antibody

NA cells cultured in 96-well plates were infected with the recombinant RABV at 37ºC for 48 h. The supernatant was removed, and the cells were fixed with 80% acetone at −20ºC for 30 min. Then, the cells were washed with PBS three times. The anti-IL-25 antibody was added and the fixed cells were incubated at 37ºC for 1 h and washed with PBS three times. The cells were then incubated with FITC-conjugated anti-rabbit IgG at 37ºC for 2 h. The cells were washed with PBS three times, and antigen-positive foci (green) were observed under a fluorescence microscope (AMG).

## The growth characteristics of RABV

Recombinant virus rHEP-IL25 and its parental virus HEP-Flury were cultured in NA cells, and their titres were determined as focus-forming units (FFU) per millilitre (FFU/mL) using the Karber method. The proliferation curves of rHEP-IL25 in NA cells and BSR cells were built. NA or BSR cells cultured in cell culture dishes were infected with virus either at a multiplicity of infection (MOI) of 0.01 or 1. Culture supernatants were collected daily for four days after inoculation. Virus titres of the collected supernatants were determined in BSR or NA cells by dFA.

## The spread of RABV

A virus spread assay was performed in NA cells as described previously [17]. Briefly, NA cells were infected with rHEP-IL25 or HEP-Flury at an MOI of 0.01 and incubated at 37ºC for 2 h. The inoculum was discarded, and the cells were lavated with PBS three times. Next, the cells were fixed with 80% acetone and washed with PBS three times. The fixed cells were inoculated with FITC-conjugated anti-RABV-N antibodies at 37ºC for 2 h. The treated cells were then stained with DAPI to display the nucleus. Fluorescence was observed under a fluorescence microscope.

## Pathogenicity of RABV in adult mice

To determine the pathogenicity of rHEP-IL25 on adult mice, 1.0 × 10^5^ FFU rHEP-IL25 or HEP-Flury was intramuscularly (i.m.) injected into adult female KM mice (8–10 mice in each group). The medium injection was used as a mock group. Body weight and clinical symptoms were recorded daily for 21 consecutive days. The data were analysed using GraphPad Prism 6 software (GraphPad Software, USA).

**Immunization and *in vivo* challenge** To observe the immunogenicity of rHEP-IL25, 6–7 weeks old female KM mice were used in this study. Groups of 20 KM mice were i.m. immunized with 1.0 × 10^5^ FFU of rHEP-IL25 or HEP-Flury, respectively, 100 µL each mouse. An equal volume of cell culture medium control group was set at the same time. Peripheral blood was collected on days 14 and 21 after immunization, and serum was used to determine VNA levels using fluorescent antibody virus neutralization (FAVN) tests as described previously [18]. The IgG1 and IgG2a in serum were determined with anti-IgG1 and anti-IgG2a antibodies using western blot as described previously [16]. Levels of IgG2c in serum were determined using Enzyme-Linked Immunosorbent Assay (ELISA) Kits (Meimian Biotechnology, Jiangsu, China) according to the manufacturer’s instructions. The mice were intracerebrally (i.c.) challenged with 1.0 × 10^5^ FFU of CVS-11 in a total volume of 30 µL for challenge experiments 21 days after immunization. The number of survived mice was recorded daily for consecutive 21 days. The survived rates were analysed using GraphPad Prism 6 software. Body weight and clinical symptoms of all the challenged mice were recorded daily for 16 days. With regards to clinical symptoms observed in mice, scores were assigned as follows: 0, no clinical symptoms observed; 1, loss of initial body weight >5% or/and piloerection; 2, paralysis, coma; 3, death. The data were analysed using GraphPad Prism 6 software.

## Results

### Identification of recombinant RABV

**Figure 1.**
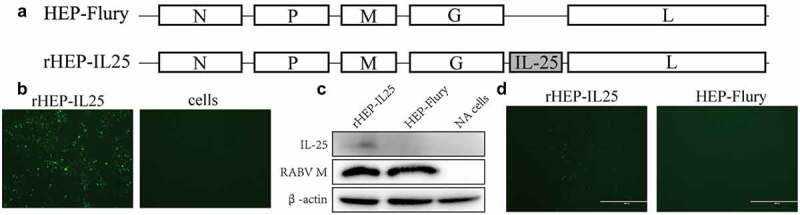
Confirmation of recombinant rHEP-IL25. (A) Schematic diagrams of the RABV genomes. N, nucleoprotein; P, phosphoprotein; M, matrix protein; G, glycoprotein; L, RNA-dependent RNA polymerase. (B) the rescued recombinant RABV expression of IL-25 was investigated by dFA with fluorescent antibody anti-RABV N antibody. (C) IL-25 expression and RABV M was determined by the western blot assay with anti-IL-25 and anti-RABV M antibodies. (D) IL-25 expression in NA cells was determined by iFA with the anti-IL-25 antibody.

Recombinant cDNA clones carrying IL-25 were constructed, and recombinant RABV was generated in BHK-21 cells. IL-25 was expressed between G and L genes, and the recombinant RABV was designated as rHEP-IL25 (Figure 1a). NA cells were inoculated with this recombinant strain to confirm the infectivity of rHEP-IL25. Green foci for antigens were confirmed by dFA (Figure 1b). In addition, the expression of RABV M or IL-25 was identificated by western blotting with anti-IL-25 and anti-RABV M antibodies. IL-25 protein was detected in NA cells infected with rHEP-IL-25 (Figure 1c). Furthermore, IL-25 expression was identificated by iFA after infection with rHEP-IL25 in NA cells (Figure 1d).

#### Not significantly effect on the RABV production *in* *vitro* by IL-25 insertion

**Figure 2.**
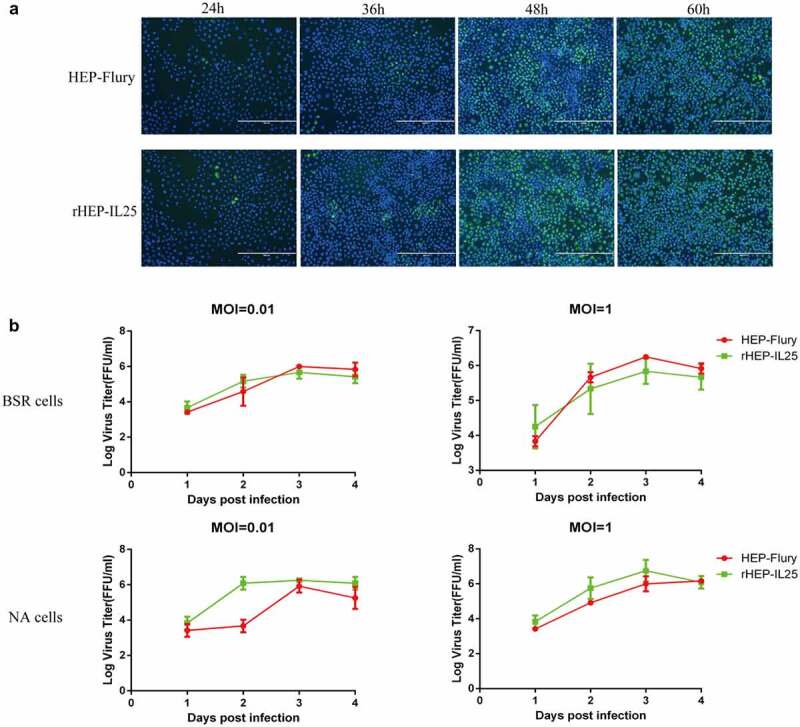
The characteristics of rHEP-IL25 *in vitro*. (A) Spread assay of RABV in cells. rHEP-IL25 or HEP-Flury were inoculated to a monolayer of NA cells at an MOI of 0.01 and followed by incubated at 37ºC. The cells were fixed with 80% acetone at 24-, 36-, 48-, and 60-hours post-infection intervals and stained with fluorescent antibody anti-RABV N protein and DAPI. (B) the growth characteristics of RABVs in BSR and NA cells. rHEP-IL25 or HEP-Flury were inoculated to BSR or NA cells at an MOI of 0.01 or 1. The supernatants of the medium were collected at 1-, 2-, 3-, and 4-day post-infection intervals and virus titres were detected.

NA cells were infected with rHEP-IL25 or HEP-Flury at an MOI of 0.01, followed to stain by fluorescent antibody to investigate whether the expression of IL-25 altered virus spread ability between cell and cell. As shown in Figure 2a, the number of the fluorescent foci (green) in the cells infected with rHEP-IL25 was not different from the cells infected with HEP-Flury. Thus, IL-25 expressed in RABV did not affect the virus spread in cells. In addition, the growth kinetics of rHEP-IL25 and parental virus HEP-Flury were investigated in NA and BSR cells infected at MOI of 0.01 and 1. As shown in Figure 2b, rHEP-IL25 exhibit the same growth curves and virus titres in both NA and BSR cells compared with the parent strain. Therefore, the expression of IL-25 did not significantly change the growth of RABV. Taken together, the overexpression of IL-25 did not influence RABV production.

#### Il-25 expression does not increase the virulence of RABV

**Figure 3.**
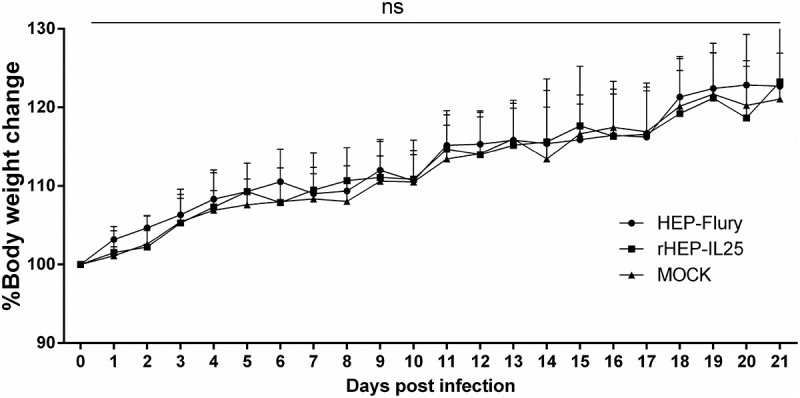
Pathogenicity of RABV in adult mice. rHEP-IL25, HEP-Flury, or medium were i.M.-inoculated to KM mice. Body weight was recorded daily for 21 days. The results showed as the body weight ratio on day 0, and data were presented as mean value ± SD (n = 8～10; ns, non-significant).

RABV HEP-Flury strain was apathogenic for adult mice and used as live vaccine before 2018 in China. To investigate whether IL-25 increased the pathogenicity of HEP-Flury, KM mice were infected intramuscularly (i.m.) with 1.0 × 10^5^ FFU of RABVs or medium (mock). The body weights and clinical symptoms of all the mice were monitored daily for 21 days. During the infection, no mice showed significant clinical symptoms of rabies. Furthermore, as shown in Figure 3, the body weight changes of mice were not significantly different between rHEP-IL25 and HEP-Flury infection. Therefore, the expression of IL-25 in RABV did not increase the virus pathogenicity.

#### IL-25 expression enhances VNA level in the periphery

**Figure 4.**
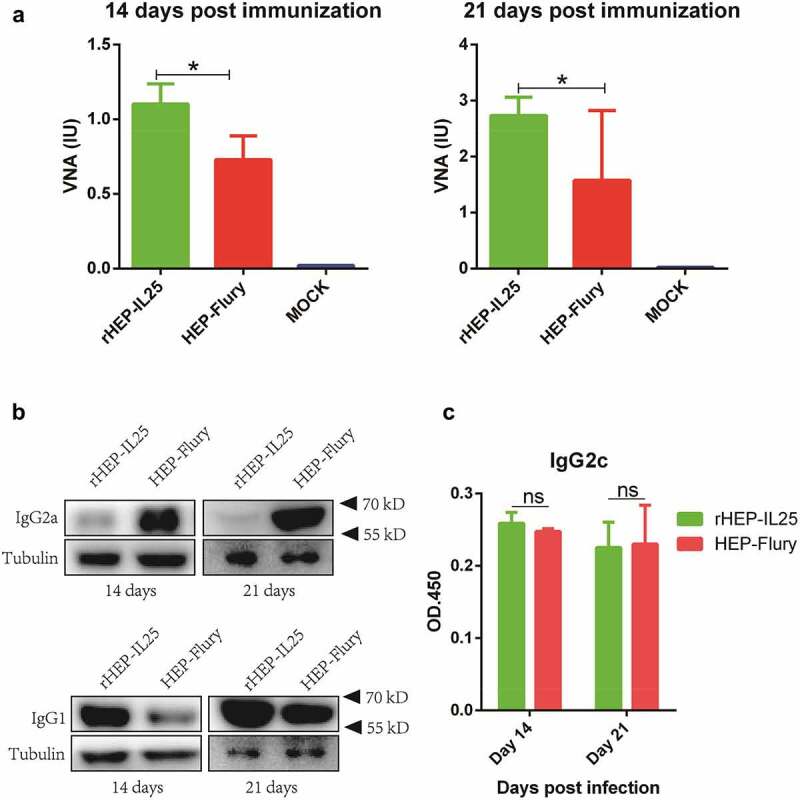
Immune response in the periphery induced by rHEP-IL25. (A) VNA levels. KM mice were i.M.-immunized with rHEP-IL25, HEP-Flury, or medium (mock infections). Peripheral blood was obtained on days 14 and 21 after immunization, and serum VNA levels were determined by FAVN. The data were presented as mean ± SD (n = 20). The data were analysed with ANOVA (*p < 0.05). (B) IgG1 and IgG2a levels. IgG1 and IgG2a in peripheral blood obtained on days 14 and 21 after immunization were determined by western blot with anti-IgG1 and anti-IgG2a antibodies. (C) IgG2c levels. IgG2c in peripheral blood obtained on days 14 and 21 after immunization was determined by ELISA assay. The results were presented as the mean of OD_450_ ± SD (n = 3). The data were analysed by the Student’s *t* test (ns, none significant).

1.0 × 10^5^ FFU of rHEP-IL25 or HEP-Flury were injected to KM mice i.m to investigate whether IL-25 improved the immune response induced by RABV. Peripheral blood samples were collected on days 14 and 21 after (post) immunization (dpi), and serum was separated to determine VNA as described above. As shown in Figure 4a, titres of VNA in the immunized mice with rHEP-IL25 or HEP-Flury were >0.5 IU at 14 and 21 dpi. Interestingly, at both 14 and 21 dpi rHEP-IL25 induced significantly higher levels of VNA in the immunized mice than HEP-Flury. These findings indicated that rHEP-IL25 enhanced more humoral immune response in mice than parent HEP-Flury.

To further investigate whether increased VNA by IL-25 expression was associated with Th2 response, IgG1 and IgG2a in serum were determined by western blot and IgG2c was determined by ELISA. As shown in Figure 4b, rHEP-IL25 induced significantly higher levels of IgG1 than HEP-Flury in serum at both 14 and 21 dpi. Less IgG2a were determined in the serum of mice infected with rHEP-IL25 than those infected with HEP-Flury (Figure 4b). The IgG2c levels were not significantly different between rHEP-IL25 and HEP-Flury infection (Figure 4c). Therefore, the expression of IL-25 in RABV might enhance Th2 activation in infected mice.

**rHEP-IL25 provides better protection against lethal RABV challenge**

**Figure 5.**
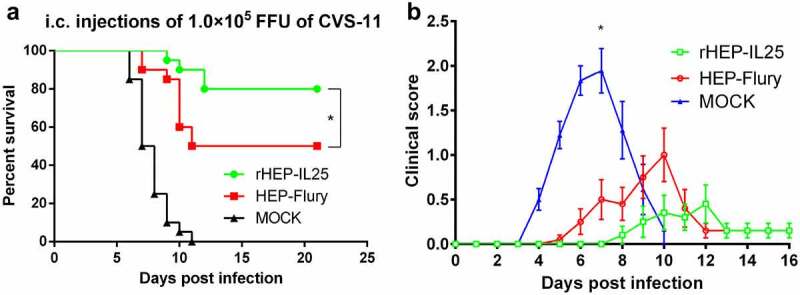
Challenged assay. Immunized mice were i.C. challenged with lethal strain CVS-11. (A)survival rates were recorded daily for three weeks. The results were presented as the mean ± SD (n = 20). The data were analysed by the log-rank Mantel-Cox test (*p < 0.05). (B) Clinical symptoms presented by challenged mice were scored according to severity on a scale from 0 to 3 and are presented here as mean values of scores ±SD (n = 18～20). Asterisks indicate significant differences among groups, as calculated by Student’s t test (*P < 0.05).

To explore the relation between the VNA titres and infection protection, the immunized mice with either rHEP-IL25 or HEP-Flury were challenged with virulent CVS-11 through i.c. infection. As shown in Figure 5a, the mice immunized with rHEP-IL25 exhibited a significantly higher survival rate than those immunized with HEP-Flury. Clinical symptoms of all challenged mice observed are shown in Figure 5b. rHEP-IL25 immunized mice showed milder clinical symptoms than those immunized with HEP-Flury after CVS-11 challenge. Thus, IL-25 expression could enhance the protective effect of the rabies vaccine.

## Discussion

Molecular adjuvant expressed by viruses should not diminish the virion production to decrease the vaccine costs. Previous studies have indicated that the pseudogene between G and L exerts no discernible effect on the virus replication of RABV [19,20]. In this study, IL-25 was expressed between G and L genes, and the recombinant RABV exhibited growth curves and replication similar to those of the parent strain. Furthermore, previous studies reported that foreign genes expressed between G and L change viral growth and replication [21–23]. However, some studies have indicated that the insertion of foreign genes does not significantly affect viral growth and replication [24–28]. Thus, different foreign proteins expressed between G and L of RABV exert different effects on viral growth and replication. Here, IL-25 did not change RABV growth and replication in NA and BSR cells.

A study showed that chemokine MIP-1α expressed between G and L significantly enhanced immune responses induced by RABV while inactivated MIP-1α did not affect the immunogenicity of recombinant RABV [29]. Thus, only the nucleotide sequence cloned into pseudogene sequence between G and L did not affect the immunogenicity of recombinant RABV. In this study, RABV expressing IL-25 between G and L induced higher IgG1, which is the product of Th2 response. Previous study indicated that IL-25 mediates Th2 activation [10]. Therefore, expression of IL-25 may contribute to the higher VNA induced by RABV.

Previous studies have shown that VNA induced by the G of RABV is critical to preventing RABV infection [30,31]. Therefore, the primary objective when we focus on developing rabies vaccines is to improve the level of VNA. In this study, for the first time, we presented evidence that IL-25 expressed in RABV could enhance the humoral immune responses induced by RABV. Furthermore, we found that IL-25 expression augmented IgG1 levels in serum. Therefore, we suspect that IL-25 improves the VNA concentration by promoting a Th2-type immune response after RABV infection [32,33]. Th2 cells could stimulate high titres of antibodies during infections [34,35]. Consistent with this, we showed that IL-25 could lift the protective effect of recombinant RABV immunization, raising the survival of the mice challenged by a RABV CVS-11 and diminishing clinical signs of rabies.

In conclusion, IL-25 expressed in RABV did not interfere with virus replication in vitro and did not increase the pathogenicity of RABV in mice. rHEP-IL25 enhanced viral immunogenicity and provided excellent immune protection against infection with virulent RABV. The findings indicated that IL-25 could enhance the humoral immune responses induced by viral vaccines. Therefore, rHEP-IL25 has the potential to be an attenuated vaccine. In addition, this study provides a reference that IL-25 can be used as a viral vaccine adjuvant.

## Data Availability

The authors confirm that the data supporting the findings of this study are available within the article or its supplementary materials.

## References

[1] Fu ZF Rabies and rabies research: past, present and future. Vaccine. 1997;15:S20–S24. 9218287 10.1016/s0264-410x(96)00312-x

[2] Schnell MJ, Mebatsion T, Conzelmann KK Infectious rabies viruses from cloned cDNA. Embo J. 1994;13(18):4195–4203. 7925265 10.1002/j.1460-2075.1994.tb06739.x PMC395346

[3] Luo J, Zhang B, Wu Y, et al. Expression of interleukin-6 by a recombinant rabies virus enhances its immunogenicity as a potential vaccine. Vaccine. 2017;35(6):938–944. DOI:10.1016/j.vaccine.2016.12.069 28089546

[4] Zhang S, Hao M, Feng N, et al. Genetically modified rabies virus vector-based rift valley fever virus vaccine is safe and induces efficacious immune responses in mice. Viruses. 2019;11(10):919. DOI:10.3390/v11100919 PMC6832564 31597372

[5] Kurup D, Malherbe DC, Wirblich C, et al. Inactivated rabies virus vectored SARS-CoV-2 vaccine prevents disease in a Syrian hamster model. PLoS Pathog. 2021;17(3):e1009383. DOI:10.1371/journal.ppat.1009383 33765062 PMC8023494

[6] Wang H, Jin H, Feng N, et al. Using rabies virus vaccine strain SRV9 as viral vector to express exogenous gene. Virus Genes. 2015;50(2):299–302. DOI:10.1007/s11262-014-1160-y 25724175

[7] Wang Z, Liang Q, Zhang Y, et al. An optimized HMGB1 expressed by recombinant rabies virus enhances immunogenicity through activation of dendritic cells in mice. Oncotarget. 2017;8(48):83539–83554. DOI:10.18632/oncotarget.18368 29137362 PMC5663534

[8] Lee J, Ho WH, Maruoka M, et al. IL-17E, a novel proinflammatory ligand for the IL-17 receptor homolog IL-17rh1. J Biol Chem. 2001;276(2):1660–1664. DOI:10.1074/jbc.M008289200 11058597

[9] Iwakura Y, Ishigame H, Saijo S, et al. Functional specialization of interleukin-17 family members. Immunity. 2011;34(2):149–162. 21349428 10.1016/j.immuni.2011.02.012

[10] Kim MR, Manoukian R, Yeh R, et al. Transgenic overexpression of human IL-17E results in eosinophilia, B-lymphocyte hyperplasia, and altered antibody production. Blood. 2002;100(7):2330–2340. DOI:10.1182/blood-2002-01-0012 12239140

[11] Fallon PG, Ballantyne SJ, Mangan NE, et al. Identification of an interleukin (IL)-25-dependent cell population that provides IL-4, IL-5, and IL-13 at the onset of helminth expulsion. J Exp Med. 2006;203(4):1105–1116. DOI:10.1084/jem.20051615 16606668 PMC2118283

[12] Owyang AM, Zaph C, Wilson EH, et al. Interleukin 25 regulates type 2 cytokine-dependent immunity and limits chronic inflammation in the gastrointestinal tract. J Exp Med. 2006;203(4):843–849. DOI:10.1084/jem.20051496 16606667 PMC1800834

[13] Luo J, Zhao J, Tian Q, et al. A recombinant rabies virus carrying GFP between N and P affects viral transcription in vitro. Virus Genes. 2016;52(3):379–387. DOI:10.1007/s11262-016-1313-2 26957093 PMC4858564

[14] Inoue K, Shoji Y, Kurane I, et al. An improved method for recovering rabies virus from cloned cDNA. J Virol Methods. 2003;107(2):229–236. DOI:10.1016/S0166-0934(02)00249-5 12505638

[15] Luo J, Zhang Y, Zhang Q, et al. The deoptimization of rabies virus matrix protein impacts viral transcription and replication. Viruses. 2019;12(1): DOI:10.3390/v12010004 PMC7019236 31861477

[16] Luo J, Zhang Y, Wang Y, et al. Rhabdovirus infection is dependent on Serine/Threonine kinase AP2-associated kinase 1. Life (Basel). 2020;10(9): DOI:10.3390/life10090170 PMC7554979 32872567

[17] Luo J, Zhang B, Lyu Z, et al. Single amino acid change at position 255 in rabies virus glycoprotein decreases viral pathogenicity. Faseb J. 2020;34(7):9650–9663. DOI:10.1096/fj.201902577R 32469133

[18] Cliquet F, Aubert M, Sagne L Development of a fluorescent antibody virus neutralisation test (FAVN test) for the quantitation of rabies-neutralising antibody. J Immunol Methods. 1998;212(1):79–87. 9671155 10.1016/s0022-1759(97)00212-3

[19] Mebatsion T, Schnell MJ, Cox JH, et al. Highly stable expression of a foreign gene from rabies virus vectors. Proc Natl Acad Sci U S A. 1996;93(14):7310–7314. 8692989 10.1073/pnas.93.14.7310 PMC38980

[20] Ceccaldi PE, Fayet J, Conzelmann KK, et al. Infection characteristics of rabies virus variants with deletion or insertion in the pseudogene sequence. J Neurovirol. 1998;4(1):115–119. 9531019 10.3109/13550289809113489

[21] Schnell MJ, Foley HD, Siler CA, et al. Recombinant rabies virus as potential live-viral vaccines for HIV-1. Proc Natl Acad Sci U S A. 2000;97(7):3544–3549. DOI:10.1073/pnas.97.7.3544 10706640 PMC16276

[22] Wang Y, Tian Q, Xu X, et al. Recombinant rabies virus expressing IFNalpha1 enhanced immune responses resulting in its attenuation and stronger immunogenicity. Virology. 2014;468-470:621–630. 25310498 10.1016/j.virol.2014.09.010

[23] Mcgettigan JP, Pomerantz RJ, Siler CA, et al. Second-Generation rabies virus-based vaccine vectors expressing human immunodeficiency virus type 1 gag have greatly reduced pathogenicity but are highly immunogenic. J Virol. 2003;77(1):237–244. DOI:10.1128/JVI.77.1.237-244.2003 12477829 PMC140592

[24] Zhang Y, Yang J, Li M, et al. A recombinant rabies virus expressing fms-like tyrosine kinase 3 ligand (Flt3l) induces enhanced immunogenicity in mice. Virol Sin. 2019;34(6):662–672. DOI:10.1007/s12250-019-00144-x 31254272 PMC6889250

[25] Xue X, Zheng X, Liang H, et al. Generation of recombinant rabies Virus CVS-11 expressing eGFP applied to the rapid virus neutralization test. Viruses. 2014;6(4):1578–1589. DOI:10.3390/v6041578 24714411 PMC4014711

[26] Zhou M, Wang L, Zhou S, et al. Recombinant rabies virus expressing dog GM-CSF is an efficacious oral rabies vaccine for dogs. Oncotarget. 2015;6(36):38504–38516. DOI:10.18632/oncotarget.5904 26436700 PMC4770717

[27] Chen T, Zhang Y, Wang Z, et al. Recombinant rabies virus expressing IL-15 enhances immunogenicity through promoting the activation of dendritic cells in mice. Virol Sin. 2017;32(4):317–327. DOI:10.1007/s12250-017-4036-1 28861771 PMC6598908

[28] Zhang Y, Zhou M, Wang Z, et al. Recombinant rabies virus expressing IL-21 enhances immunogenicity through activation of T follicular helper cells and germinal centre B cells. J Gen Virol. 2016;97(12):3154–3160. DOI:10.1099/jgv.0.000646 27902338

[29] Zhao L, Toriumi H, Wang H, et al. Expression of MIP-1alpha (CCL3) by a recombinant rabies virus enhances its immunogenicity by inducing innate immunity and recruiting dendritic cells and B cells. J Virol. 2010;84(18):9642–9648. DOI:10.1128/JVI.00326-10 20592092 PMC2937656

[30] Koraka P, Bosch BJ, Cox M, et al. A recombinant rabies vaccine expressing the trimeric form of the glycoprotein confers enhanced immunogenicity and protection in outbred mice. Vaccine. 2014;32(36):4644–4650. DOI:10.1016/j.vaccine.2014.06.058 24962755

[31] Liu X, Yang Y, Sun Z, et al. A recombinant rabies virus encoding two copies of the glycoprotein gene confers protection in dogs against a virulent challenge. PLoS One. 2014;9(2):e87105. DOI:10.1371/journal.pone.0087105 24498294 PMC3911940

[32] Wang C, Liu Q, Chen F, et al. IL-25 promotes Th2 immunity responses in asthmatic mice via nuocytes activation. PLoS One. 2016;11(9):e162393. 10.1371/journal.pone.0162393 PMC5019461 27617447

[33] Wang H, Jiang Y, Wang H, et al. IL-25 promotes Th2-type reactions and correlates with disease severity in the pathogenesis of oral lichen planus. Arch Oral Biol. 2019;98:115–121. 30472360 10.1016/j.archoralbio.2018.11.015

[34] George JA, Kim SB, Choi JY, et al. Tlr2/myd88 pathway-dependent regulation of dendritic cells by dengue virus promotes antibody-dependent enhancement via Th2-biased immunity. Oncotarget. 2017;8(62):106050–106070. DOI:10.18632/oncotarget.22525 29285314 PMC5739701

[35] Spellberg B, Edwards JJ Type 1/type 2 immunity in infectious diseases. Clin Infect Dis. 2001;32(1):76–102. 11118387 10.1086/317537

